# Laboratory test result interpretation for primary care doctors in South Africa

**DOI:** 10.4102/ajlm.v6i1.453

**Published:** 2017-03-24

**Authors:** Naadira Vanker, Norman H. B. Faull

**Affiliations:** 1 Graduate School of Business, University of Cape Town, Cape Town, South Africa

## Abstract

**Background:**

Challenges and uncertainties with test result interpretation can lead to diagnostic errors. Primary care doctors are at a higher risk than specialists of making these errors, due to the range in complexity and severity of conditions that they encounter.

**Objectives:**

This study aimed to investigate the challenges that primary care doctors face with test result interpretation, and to identify potential countermeasures to address these.

**Methods:**

A survey was sent out to 7800 primary care doctors in South Africa. Questionnaire themes included doctors’ uncertainty with interpreting test results, mechanisms used to overcome this uncertainty, challenges with appropriate result interpretation, and perceived solutions for interpreting results.

**Results:**

Of the 552 responses received, the prevalence of challenges with result interpretation was estimated in an average of 17% of diagnostic encounters. The most commonly-reported challenges were not receiving test results in a timely manner (51% of respondents) and previous results not being easily available (37%). When faced with diagnostic uncertainty, 84% of respondents would either follow-up and reassess the patient or discuss the case with a specialist, and 67% would contact a laboratory professional. The most useful test utilisation enablers were found to be: interpretive comments (78% of respondents), published guidelines (74%), and a dedicated laboratory phone line (72%).

**Conclusion:**

Primary care doctors acknowledge uncertainty with test result interpretation. Potential countermeasures include the addition of patient-specific interpretive comments, the availability of guidelines or algorithms, and a dedicated laboratory phone line. The benefit of enhanced test result interpretation would reduce diagnostic error rates.

## Introduction

Laboratory services play an integral role in the healthcare system - from primary- through to tertiary-level care – since diagnostic tests can either confirm or exclude a tentative diagnosis, or screen for potential diseases.^1^ The underlying purpose of laboratory testing lies in the association between laboratory test results and the potential to improve a patient’s health status. This requires ordering a test when appropriate and necessary, accurate interpretation, and acting upon the result.^2^

Primary care doctors are usually the point of entry into a healthcare system, and are consequently exposed to a variety of medical conditions that range in both complexity and severity. It is postulated that these doctors are therefore at a higher risk of making medical errors than specialists.^3^ Due to the variety and intricacies of laboratory tests available, there is the potential for test-related errors to occur in a range of clinical conditions, that may result in significant patient harm.^4^ Studies have shown that between 15% and 54% of errors occurring at a primary healthcare level are related to the testing process.^5^ Diagnostic errors can be due to three underlying causes, namely: no identifiable fault, system-related, and cognitive. Cognitive errors are caused by incorrect interpretation of available information and may be caused by faulty knowledge, faulty data gathering, or faulty synthesis of data. A large-scale study found that up to 74% of diagnostic errors are either completely or in part due to cognitive failures.^6^ This suggests that many diagnostic errors are related to misunderstanding or misinterpreting the available information.

Studies have shown that primary care doctors face uncertainty when interpreting clinical laboratory reports.^7,8,9,10^ A nationwide study involving 1768 primary care physicians was conducted in the United States to determine the challenges that this group faces with laboratory test ordering and result interpretation. The findings were that clinicians experienced uncertainty due to inconsistencies in the receipt of results, problems with the report format, and difficulties with interpretation of results in 8.3% of diagnostic encounters.^7^ Research conducted in South Africa also demonstrated the uncertainty that doctors experience with laboratory test result interpretation. This study investigated how confident interns were with requesting biochemical tests and interpreting the results. The study found that although these junior doctors were fairly confident when dealing with common investigations, they experienced challenges with interpreting the results of more complex and less common tests. Of the 61 respondents, 23% reported a lack of confidence in interpreting the results of complex tests.^8^

This explorative study investigated the problems and challenges that primary healthcare doctors in South Africa face with the interpretation of clinical laboratory test results. A secondary aim of the study was to identify potential countermeasures to address these challenges.

## Methods

### Ethical considerations

Ethical approval was granted by the Commerce Faculty Ethics in Research Committee, Graduate School of Business, University of Cape Town. Anonymity was maintained throughout the process. Informed consent was obtained through a cover letter containing the survey link. The surveys were completed online, with all responses anonymised through the system. The researchers did not have access to respondents’ identifying or personal information.

### Study design

This research was based on a study conducted by Hickner et al., entitled ‘Primary care physicians’ challenges in ordering clinical laboratory tests and interpreting results’.^7^ The original survey was developed through an inductive approach, using information obtained from three focus groups comprising 27 primary care doctors, as well as from a panel of experts working in primary healthcare and laboratory medicine. The questionnaire was authorised for use by the Office of the Associate Director for Science at the US Centers for Disease Control and Prevention.

The original nineteen-part questionnaire was reduced to nine sections to focus on the challenges that primary healthcare doctors face with clinical laboratory result interpretation. The survey themes included doctors’ uncertainty with interpreting test results, mechanisms they use to overcome this uncertainty, challenges with appropriate result interpretation, and perceived solutions to interpreting test results. The questionnaire categories were as follows: (1) demographic information; (2) information about the doctor’s practice; (3) interpretation uncertainty; (4) the diagnostic evaluation process; (5) laboratory consultation; and (6) test utilisation enablers. Questions related to the doctor’s practice included: whether the doctor was a general practitioner or specialist; the number of years in practice; the predominant categories of tests ordered (i.e. diagnostic tests, chronic disease monitoring, or routine screening); the number of patients seen per week; the number of tests ordered per week; and the number of tests per week that were associated with interpretation uncertainty. Two questions were added to the demographic section of the questionnaire to determine whether the South African doctor worked in a rural, semi-urban or urban practice and whether he/she predominantly made use of private pathology laboratories or the parastatal (National Health Laboratory Service) laboratory. However, it was not ascertained whether the majority of patients seen were hospitalised or out-patients. Responses were predominantly chosen from a list of five-point graded options, but there was space given for open-ended responses. Response options ranged from: ‘extremely useful’ to ‘not at all useful’, ‘extremely important’ to ‘not at all important’, ‘extremely well’ to ‘not at all well’, and ‘extremely problematic’ to ‘not at all problematic’.

### Survey administration

For this cross-sectional study, questionnaires were sent out electronically using a survey link to the approximately 7800 primary care doctors in the South African Medical Association database. The survey was sent on 13 October 2015 and remained open for responses until 13 November 2015.

### Analysis

Response data from the surveys were exported to Microsoft Excel (Microsoft Corp., Redmond, Washington, United States) and analysed using the IBM SPSS Statistics® package (IBM SPSS Statistics for Macintosh, Version 22.0.; IBM Corp., Armonk, New York, United States). The qualitative responses were analysed quantitatively using descriptive statistics to determine relative frequencies. Results presented were based on the number of respondents who selected the top two responses from the five-point scale – namely, ‘extremely and very useful’, ‘extremely and very important’, ‘extremely and very well’, or ‘extremely and very problematic’. Open-ended responses received were reported as ‘other’ in the figures below.

## Results

### Overview and respondent characteristics

Of the approximately 7800 questionnaires sent out, 552 completed questionnaires were received, equating to a response rate of 7%. Incomplete questionnaires were excluded from the analysis, so as not to skew the results. Although the survey was sent to doctors registered in the South African Medical Association database as general and/or independent practitioners, this database included a few doctors who were either in training or were qualified specialists. Table 1 describes the doctors’ practice characteristics and test utilisation information. Of note, respondents saw an average of 115 patients per week, ordered an average of 24 tests per week, and experienced uncertainty in result interpretation for four of these tests. This equates to challenges in the interpretation of approximately 17% of test results.

**TABLE 1:** Respondents’ practice characteristics and laboratory test utilisation information, South Africa, 13 October 2015–13 November 2015.

Variables	Number of respondents	Result	Range
Average number of years in practice	552	13 years	1–47 years
General or specialist practitioner			
General Practitioner	513	93%	-
Specialist^†^	39	7%	-
Location of practice			
Urban	298	54%	-
Peri-urban	133	24%	-
Rural	121	22%	-
Type of laboratory utilised most frequently			
Government/Parastatal (National Health Laboratory Service)	304	55%	-
Private	248	45%	-
Predominant categories (50–100% of tests) ordered by individual doctors			
Diagnostic tests	248	45%	-
Chronic disease monitoring	138	25%	-
Routine screening	94	17%	-
No predominance	72	13%	-
Average number of patients seen per week	552	115 patients	3–500 patients
Average number of laboratory tests ordered per week	552	24 tests	0–200 tests
Average number of tests per week for which there is uncertainty in result interpretation	552	4 tests	0–30 tests

† Fields of specialty included: family medicine, internal medicine, emergency medicine, palliative medicine, HIV care, sports medicine, psychiatry, anaesthetics, paediatrics, public health, general surgery, and neurosurgery.

### Challenges with laboratory test results

**FIGURE 1:**
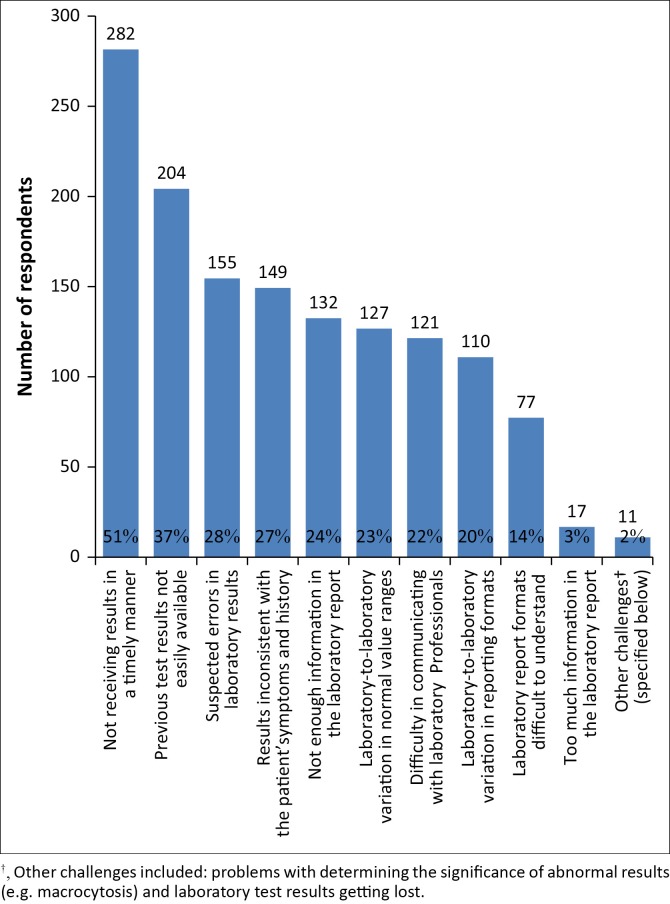
Challenges that doctors face when using laboratory test results, South Africa, 13 October 2015–13 November 2015.

The challenges that doctors experienced with laboratory test results (Figure 1) were selected from a list of possible options and rated using the five-point scale. The most prominent problems were related to accessing results with 51% of respondents reporting that not receiving results in a timely manner was very problematic and 37% reporting problems with availability of previous test results. (The timeliness of the receipt of results was as perceived by the respondents and was not quantified or defined in the question.) The next-highest reported type of challenge related to the result being incompatible with the patient’s clinical picture, which could either be seen as an inconsistent result (27%) or a laboratory error (28%).

Although not strictly related to challenges with laboratory test results, a few respondents did report challenges with laboratory access and financing in the open-ended part of this section. These challenges included: difficulties for rural hospitals or practices to get samples to a laboratory, the unavailability of specialised tests (e.g., B-type natriuretic peptide and *Helicobacter pylori* IgG), and medical aids not authorising or paying for tests.

### Diagnostic evaluation process

The majority of doctors (66%) typically used a core set of 20 or fewer clinical laboratory diagnostic tests. When faced with diagnostic uncertainty, most respondents (67%) reported always double-checking with another doctor or electronic resources (e.g., UpToDate, WebMD, patient.co.uk, etc.) if they doubted their decision. Even when confident in their pre-test diagnoses, 42% of doctors would still think ‘what else could it be?’. While 58% of clinicians were concerned about over-testing their patients, only 33% were concerned about under-testing patients.

### Interpretation uncertainty

**FIGURE 2:**
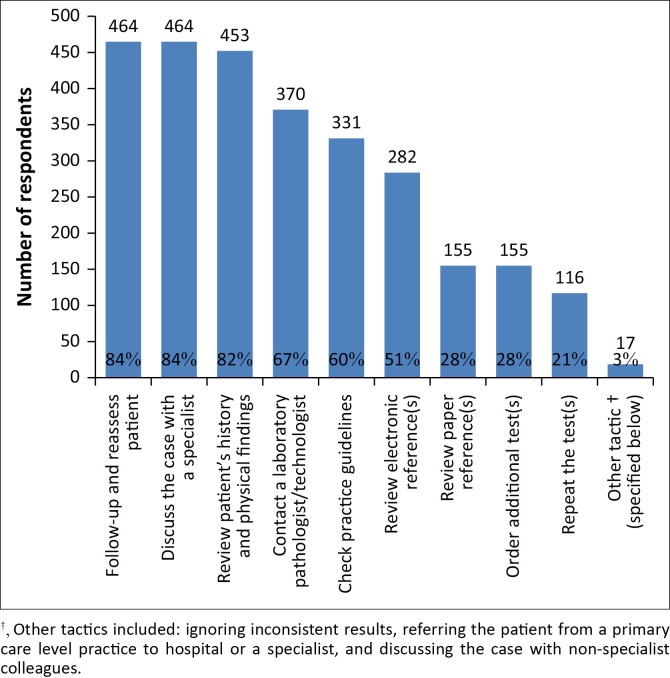
Tactics employed by doctors to deal with test interpretation uncertainty, South Africa, 13 October 2015–13 November 2015.

When faced with diagnostic uncertainty in a difficult or unusual case (Figure 2), most respondents would either follow-up and reassess the patient (84%) or review the patient’s history and physical findings (82%). Eighty-four percent of primary care doctors also found it very useful to discuss the case with a specialist.

### Laboratory consultation

**FIGURE 3:**
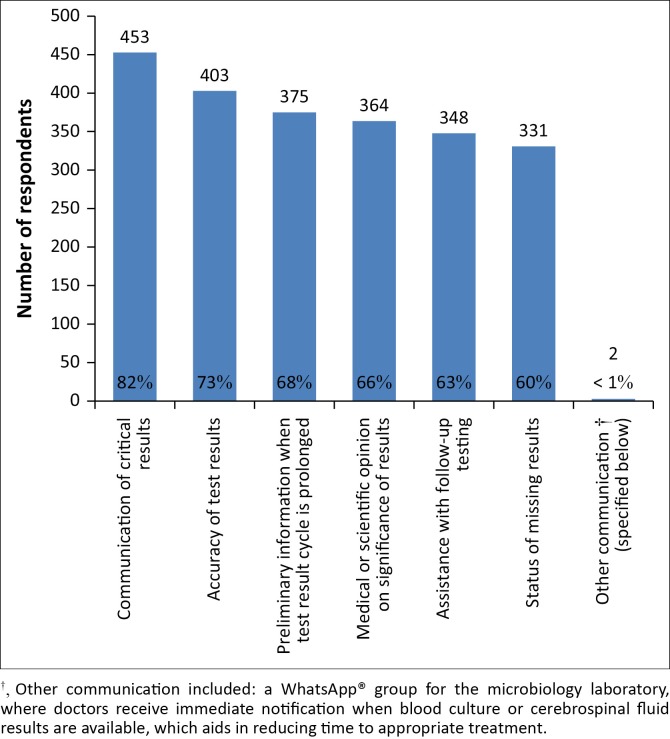
Usefulness of laboratory communication/consultation, South Africa, 13 October 2015–13 November 2015.

In a variety of contexts, most respondents found communication with the laboratory to be useful (Figure 3). Of note, 82% of doctors found it very useful for the laboratory to contact the clinician with critically abnormal results.

In general, only about one-third of respondents noted very important reasons as to why they did not frequently contact laboratory professionals. These reasons included: difficulties in contacting the person who could answer their questions (36%); not knowing whom to contact (33%); difficulties in getting through to the laboratory (32%); and not having a working relationship with laboratory professionals (27%). Only 11% reported that they did not contact the laboratory because they felt that they had received unreliable information during previous interactions. A small number (2%) of individuals reported specific problems with public sector laboratories wherein they felt that laboratory staff were unhelpful regarding lost or rejected specimens, and inaccurate, delayed or urgent results. A lack of access to pathologists at certain regional laboratories was also noted as a problem.

### Test utilisation enablers

**FIGURE 4:**
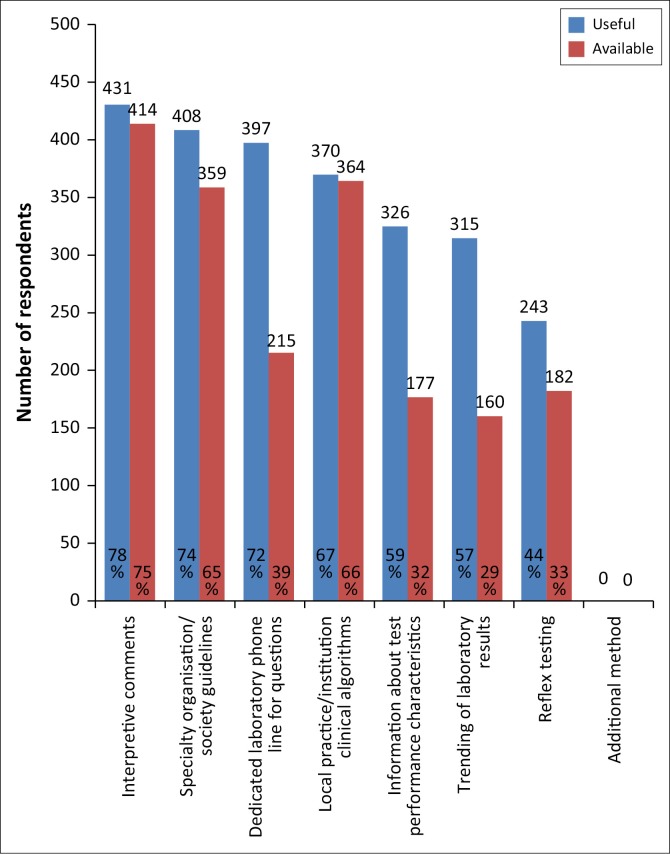
Usefulness and availability of test utilisation enablers, South Africa, 13 October 2015–13 November 2015.

Test utilisation enablers (Figure 4) are methods that have been developed to assist clinicians in using diagnostic laboratory testing more effectively. Interpretive comments – comments provided with the test result to give additional information on the meaning of the results – were reported by 78% of respondents as very useful. Seventy-four percent of doctors found guidelines – aids published by specialty organisations or societies for the interpretation of patient’s test results based on clinical presentation usually guided by decision trees – to be useful. Similarly, clinical algorithms – guidelines used within local practices or institutions – were reported as very useful by 67% of doctors. Seventy-two percent of the respondents reported that a dedicated laboratory phone was useful; however, only 39% of respondents had access to a dedicated laboratory phone line. Information on test performance characteristics, such as sensitivity, specificity, and likelihood ratios, were also reported as very useful by 59% of respondents, but were only available to 32%. The test utilisation enablers considered useful were selected from the options presented in the five-point scale questions, and no additional enablers were suggested by respondents in the open-ended response section.

When compared to urban and peri-urban respondents, rural doctors experienced considerably lower availability of interpretive comments, information on test performance characteristics, trending of laboratory results (when previous results are compared with current results), and reflex testing (a test performed by the laboratory in response to results from a previous test) (Table 2). In contrast , the rural doctors reported increased availability of a dedicated laboratory phone line.

**TABLE 2:** Availability of test utilisation enablers for the urban, peri-urban and rural doctor cohorts,† South Africa, 13 October 2015–13 November 2015.

Test Utilisation Enablers	Average ± Standard Deviation		Urban Doctors		Peri-urban Doctors		Rural Doctors	
	*N*	%	*N*	%	*N*	(%)	*N*	(%)
Interpretive comments	414 ± 39	75 ± 7	238	80	102	77	79	65
Specialty organisation/society guidelines	359 ± 11	65 ± 2	194	65	84	63	81	67
Dedicated laboratory phone line for questions	215 ± 50	39 ± 9	110	37	49	37	63	52
Local practice/institution clinical algorithms	364 ± 39	66 ± 7	200	67	97	73	71	59
Information on test performance characteristics	177 ± 33	32 ± 6	98	33	49	37	31	26
Trending of laboratory results	160 ± 28	29 ± 5	95	32	40	30	27	22
Reflex testing	182 ± 28	33 ± 5	107	36	44	33	32	26

† The cohorts consisted of 298 (54%) urban, 133 (24%) peri-urban and 121 (22%) rural medical practices.

### Further comments

Open-ended feedback on result interpretation was also elicited from respondents. Results included acknowledgement that the ‘interpretation of results are [sic] critical to reliably apply the blood results to our patients in terms of diagnosis, screening and monitoring their pathology’. A number of respondents felt that the interpretive comments currently received were not specific to patients’ age, sex, clinical picture or previous results, but were instead based on general information. Furthermore, these comments did not include recommendations for further testing or treatment. Specific challenges were noted in the interpretation of microbiology, serology (especially hepatitis B), and discordant HIV results. In contrast to the challenges noted, certain private laboratories were identified and commended for the inclusion (where necessary) of interpretive comments written by pathologists.

## Discussion

This study found that primary care doctors in South Africa experience challenges with laboratory test result interpretation in approximately 17% of their diagnostic encounters. By comparison, a similar study conducted in the United States found that primary care physicians reported uncertainty in 8.3% of diagnostic encounters. This emphasises the need for improved mechanisms and countermeasures to aid South African doctors with result interpretation.

The most common general challenges with laboratory test results reported by primary care doctors are related to receiving and accessing results – namely, not receiving results timeously and previous results not being easily available. Literature shows that over 80% of laboratories receive complaints about turn-around times,^11^ yet there are no universal evidence-based goals for laboratory processing times, and clinicians’ expectations have often been found to be unreasonable. Nevertheless, laboratories should acknowledge customer dissatisfaction, and aim to provide results within a timeframe that is achievable by the laboratory and optimal for patient care.^11^ Trending of results is the displaying of a patient’s previous results alongside the current test result to identify patterns of change and to enhance result interpretation. Over half of all respondents in our study found this to be an extremely or very useful test utilisation enabler, but less than a third reported its availability. Result trending has been shown to decrease time spent by clinicians on a case.^12^ Laboratories can play a role in addressing these challenges by determining appropriate turn-around times and communicating these times to the doctors, as well as by including previous test results on current reports to enable result trending. However, the availability of previous results requires integrated health information technology systems, which are not available in all healthcare environments.^12^

Although this study focused on the post-analytical phase of laboratory testing, the survey raised two questions around the analytic testing process and whether the clinicians experienced challenges with ‘suspected errors in laboratory results’ and ‘results inconsistent with the patient’s symptoms and history’. Inconsistent results were reported as a challenge by 27% of respondents and possible laboratory errors by 28%. However, this did not include inquiry into errors occurring in the pre-analytical phase of testing, such as the mislabeling of samples. It has been found that pre-analytical errors account for 55% of laboratory errors causing a missed or delayed diagnosis.^13^ Therefore, clinicians should be aware that suspected errors or inconsistent results might be due to failures that occur outside the control of the laboratory.

Our study found that primary care doctors find consultation with other clinicians or laboratory professionals to be an important mechanism in aiding test result interpretation. The majority of respondents reported a dedicated laboratory phone line to be an important test utilisation enabler, and, although this was only available to less than 40% of the total study population, over half of the rural doctor cohort had access to this service. This suggests that laboratories based in rural areas are trying to leverage their limited resources. A review of literature found that failures in communication between clinicians and the laboratory could negatively impact patient safety.^5^ Improving communication channels between the laboratory and clinical practitioners could lead to improved patient care and reduce unnecessary specialist referrals, which are at times requested purely for test result interpretation.^7^

The majority of survey respondents reported interpretive comments to be the most useful test utilisation enabler. Interpretive comments are added to a laboratory report in order to provide further information on the result and to aid in the diagnostic process. These comments can be provided by a qualified pathologist through technology-based interpretive algorithms and expert systems or through the addition of a ‘canned’ comment. A ‘canned’ comment is pre-written text that is added onto all results for a specific test, regardless of the actual result or the patient’s clinical history, and is considered to be the least useful form of interpretive commenting.^14^ Respondents in our study supported this view when noting that interpretive comments that were not specific to the patient’s age, sex, clinical picture or previous results, were not particularly useful. A study assessing the impact of narrative interpretations for complex laboratory tests found that the comments reduced the time taken and the number of tests required to reach a diagnosis and had an impact on the differential diagnosis. Furthermore, most respondents in that study felt that the interpretive comments helped prevent a misdiagnosis.^15^ The provision of high quality, patient-specific interpretive comments should improve patient care, decrease diagnostic errors, reduce costs, and enhance appropriate specialist referrals.^16^

The majority of respondents also reported guidelines or algorithms to be useful test-utilisation enablers. Clinical algorithms and practice guidelines are developed to provide a standardised, evidence-based approach to clinical processes in order to reduce error rates, improve clinical effectiveness, and enhance the quality of patient care.^17^ Clinical algorithms are particularly helpful in the interpretation of results for conditions that require a complex panel of tests for diagnosis, management, and monitoring of disease progression (e.g., diabetes mellitus).^18^ However, the availability of guidelines does not always ensure their use or result in changes in medical practices and behaviours. It is recommended that guidelines be disseminated through systems or accompanied by tools to facilitate their use and effectiveness.^19^ A study that compared the use of a technology-based expert system with conventional (non-computer-based) guidelines, found that the computer-based guideline system shortened the time taken to reach a diagnosis from (on average) 3.2 days to one day.^20^ The availability of guidelines or algorithms would be a useful countermeasure to aid doctors in interpreting complex tests and recommending further investigations that would guide management decisions. These guidelines may be integrated into the existing health information technology system, which may not be developed in certain settings, or can be in the form of applications that are uploaded onto independent mobile devices.^7^

It has been found that clinicians are often unaware of whether their diagnoses at the time when they are making them are correct or erroneous.^21^ Therefore, interventions to reduce errors, such as medical decision support systems, should be embedded in a system rather than being made available only when perceived to be needed.^21^ To standardise quality and improve efficiency, particularly in areas where human capital is limited, information technology can be leveraged. Studies have shown that health information technology can enhance delivery of care, reduce errors, and decrease utilisation of potentially inappropriate care.^12,19,20^ Twenty-two per cent of respondents work in rural areas and the availability of test utilisation enablers (including interpretive comments) in these areas is lower compared to urban and peri-urban areas. Furthermore, it was reported that certain regional laboratories lack access to a pathologist. In these cases, embedded technology-based solutions (such as expert systems for interpretive comments or integrated guidelines) may be particularly useful in assisting primary care doctors with test result interpretation.

### Limitations

Response rates to surveys are reported to be 10%–20%,^22^ but the response rate in our study was 7%. This could be because the electronic platform used for the survey administration dissuaded doctors uncomfortable with the technology from participating; additionally, the length of time required to complete the survey (15 minutes) may have been considered too long.^23^ However, the study by Hickner et al.,^7^ on which our study is based, had a response rate of 5.6%, suggesting that this type of research may be associated with low response rates. A possible further limitation in this study is that the challenges faced by clinicians with result interpretation may have been under-reported. Research has shown that medical doctors have a tendency to display overconfidence, which can impact self-reported findings.^24^

### Conclusion

Primary care doctors in South Africa acknowledge that they experience uncertainty when interpreting certain clinical laboratory test results. The most useful countermeasures and mechanisms identified by the doctors to improve this included: the addition of patient-specific interpretive comments; the availability of national or international guidelines or local clinical algorithms; and enhanced communication with the laboratory through a dedicated phone line. The ultimate benefit of enhanced test result interpretation would be reduced diagnostic error rates and a more efficient and effective primary healthcare system, which would reduce the rates of referral for secondary and tertiary levels of care.
